# Comparison of differences in bone microarchitecture in adult- versus juvenile-onset type 1 diabetes Asian males versus non-diabetes males: an observational cross-sectional pilot study

**DOI:** 10.1007/s12020-020-02480-5

**Published:** 2020-09-11

**Authors:** Lingling Xu, Jie Yu, Ou Wang, Yanfang Hou, Wei Li, Huabing Zhang, Fan Ping, Qun Xu, Yuxiu Li, Weibo Xia

**Affiliations:** 1 Department of Endocrinology, Key Laboratory of Endocrinology of National Health Commission, Peking Union Medical College Hospital, Chinese Academy of Medical Science, 100730 Beijing, China; 2 Department of Epidemiology and Biostatistics, Institute of Basic Medical Sciences, Chinese Academy of Medical Sciences and School of Basic Medicine, Peking Union Medical College, 100005 Beijing, China

**Keywords:** Type 1 diabetes, Bone mineral density, Bone microarchitecture, Fracture risk

## Abstract

**Purpose:**

Evidence about bone microarchitecture in Asian type 1 diabetes (T1D) patients is lacking. We assessed the bone microarchitecture in T1D patients versus controls and compare the differences between juvenile-onset and adult-onset T1D patients.

**Methods:**

This cross-sectional study recruited 32 Asian males with T1D and 32 age-, sex-, and body mass index (BMI)-matched controls. Dual-energy X-ray absorptiometry (DXA) and high-resolution peripheral quantitative computed tomography (HR-pQCT) for ultradistal nondominant radius and tibia were performed. The data were analyzed using Student’s *t* test and analysis of covariance.

**Results:**

Among the patients, 15 had juvenile-onset T1D, with a median disease duration of 11 years, and 17 had adult-onset T1D, with a median disease duration of 7 years. At the radius, adult-onset and juvenile-onset T1D patients had lower total volumetric bone mineral density (vBMD), trabecular vBMD, trabecular bone volume fraction (BV/TV), and trabecular thickness (Tb.Th) (*p* < 0.05) than the control subjects. After adjusting for BMI, disease duration, and insulin dose, juvenile-onset patients tended to have lower trabecular vBMD, BV/TV, Tb.Th, and intracortical porosity (Ct.Po) than adult-onset patients. At the tibia, adult-onset patients displayed lower total vBMD, lower Ct. vBMD, and higher Ct.Po (*p* < 0.05), while juvenile-onset patients had lower Tb.Th and standard deviation of trabecular number (1/Tb.N.SD) (*p* < 0.05) than control subjects. After adjustment for covariates, adult-onset patients tended to have higher cortical pore diameter (Ct.Po.Dm) than juvenile-onset patients.

**Conclusions:**

T1D patients were associated with compromised bone microarchitecture, adult-onset and juvenile-onset T1D patients demonstrated some differences in cortical and trabecular microarchitecture.

## Introduction

Patients with type 1 diabetes mellitus (T1D) have a substantially increased risk of fractures [1–3]. A systematic review and meta-analysis of observational studies suggested that the risk of fractures among men and women with T1D was two and four times higher than that among those without T1D, respectively [4]. Given that childhood and adolescence are critical developmental periods for bone mass accrual as well as bone geometry and microarchitecture modeling, impaired bone metabolism during this period may compromise peak bone mass and bone microarchitecture [5–8], thereby leading to the risk of fractures beginning in childhood in T1D patients and extending throughout their lifespan [1].

However, area bone mineral density (aBMD) in the hip, lumbar, and forearm as measured on dual-energy X-ray absorptiometry (DXA) is only moderately reduced (or even normal) for T1D patients compared to control subjects [9–12], which does not explain the pronounced elevated fracture risk in T1D patients [13]. Studies on volumetric BMD (vBMD) assessed using quantitative computed tomography (QCT), peripheral quantitative computed tomography (pQCT), and high-resolution pQCT (HR-pQCT) have shown that T1D patients have lower trabecular or cortical vBMD, or both, than control subjects [14]. These QCTs can also be used to evaluate bone geometry and microarchitecture, which are factors considered to be associated with fracture risk [15, 16].

Most studies have found that T1D patients have smaller cortical cross-sectional areas (CSA) and thinner cortical thicknesses (Ct.Th) than control subjects [14]. In a recent study, T1D patients were found to have higher cortical porosity measured on HR-pQCT [17]. However, only one of these studies has focused on an Asian population, and that study had a very small sample size. In that cross-sectional study conducted among Japanese subjects, 17 young or middle-aged T1D patients were found to have significantly lower cortical thickness and cortical CSA in the femoral intertrochanter than 18 healthy control subjects [18].

Studies on trabecular microarchitecture and quality in T1D patients are scarce, and their results are conflicting. Using trabecular bone score (TBS) based on DXA, three cross-sectional studies have found T1D patients to have decreased TBS values in the lumbar spine, while another study found that only T1D patients with prevalent fractures had lower TBS values [14]. HR-pQCT provides high-resolution measurement and evaluation of the trabecular microarchitecture, generating more precise trabecular bone volume (Tb.BV/TV), trabecular number (Tb.N), trabecular thickness (Tb.Th), and trabecular separation (Tb.Sp) measurements. Thus far, only three cross-sectional studies have reported HR-pQCT-related trabecular microarchitectures in T1D patients [17, 19, 20]. In 2015, Shanbhogue et al. first reported that only T1D adults with diabetic microvascular disease (MVD) had lower Tb.BV/TV than control adults, while those without diabetic microvascular disease did not [20]. In 2016, Starup-Linde et al. compared T1D and T2D patients and did not find any significant difference in the trabecular microarchitecture between the two groups [19]. Recently, a study found that teenage girls with T1D and HbA1c > 8.5% had fewer plate-like and axially aligned trabeculae than control subjects [17]. There are no studies reporting HR-pQCT related parameters in Asian T1D patients.

In addition, factors such as age, disease duration, glucose control, hypoglycemia, and diabetic chronic complications may also predispose T1D patients to an increased fracture risk [1, 21, 22]. A patient’s age at the onset of diabetes may also have an important effect on this risk. One study found that the onset of T1D before 20 years of age was associated with an increased fracture risk [23]. The underlying mechanisms remain unclear, but bone microarchitecture may be an independent predictor of incident fracture risks [15]. A study conducted among 20 T1D patients has found more prominent deficits in vBMD and cortical thickness in patients with disease onset at younger age [24]; therefore, the age at onset of T1D may be assumed to affect bone microarchitecture. However, that study recruited only female patients and did not assess the trabecular microarchitecture.

To better clarify bone damage in T1D patients across different ethnicities and to provide data in an Asian population, we aimed to study DXA and HR-pQCT related parameters in Asian T1D patients. Herein, we reported our preliminary results by comparing 32 adult male T1D patients and control subjects. Subsequently, to explore the effects of age at disease onset, we further conducted comparisons between adult-onset T1D patients and control subjects, juvenile-onset T1D patients and control subjects, and adult-onset T1D patients and juvenile-onset T1D patients.

## Materials and methods

### Study design

This was a single-center, cross-sectional study conducted in 2018. Male T1D patients aged 18 years or older (*n* = 32) were recruited from a diabetes summer camp held at Peking Union Medical College Hospital outpatient clinics in 2018. All patients had medical records meeting the diagnostic criteria for T1D. The inclusion criteria included antibodies positive for glutamic acid decarboxylase, C-peptide <700 pmol/L, and the need for an insulin regimen from the time of diagnosis. The exclusion criteria were as follows: (1) impaired renal function (estimated glomerular filtration rate <60 mL/min/1.73 m^2^); (2) history of hepatic dysfunction, malignant tumor, parathyroid disease, or other endocrine disease thought to affect bone metabolism; (3) use of medications such as glucocorticoids, thiazolidinedione, anti-osteoporotic medications, or warfarin in the past three months before enrollment; (4) smoking and drinking abuse. Control subjects without a history of chronic disease (*n* = 32) and who were apparently healthy were selected from a previously studied control cohort recruited from the community to establish a normal reference range of HR-pQCT related parameters. They were matched with the included T1D patients for age, sex, and body mass index (BMI), but only HR-pQCT-related data were obtained.

This study was approved by the Ethics Committee of the Peking Union Medical College Hospital and was in compliance with the Declaration of Helsinki. Informed consent was obtained from all patients prior to participation, and all subjects signed an informed consent document.

### Clinical and biochemical assessments

After the patients fasted for 8–12 h overnight, their urine specimens and blood samples were collected. Plasma glucose levels were measured using the glucose oxidase method. Hemoglobin A1c (A1C) was measured using HPLC (Bio-Rad D10 hemoglobin testing system). The urinary albumin-to-creatinine ratio (uACR) was measured using routine automated laboratory methods. Serum calcium, serum phosphorus, and alkaline phosphatase levels (ALP) were measured using automated analyzers. An automated Roche electrochemiluminescence system (E170; Roche Diagnostics, Basel, Switzerland) was used to measure serum levels of cross-linked C-telopeptide of type I collagen (β-CTX) and 25-hydroxy vitamin D (25(OH)D). In addition, serum testosterone levels were measured using the chemiluminescence method.

After blood and urine samples were collected, all patients completed a standardized questionnaire administered by trained interviewers. Data collected during interviews included each patient’s current age, age at onset of diabetes, smoking status and alcohol intake, fracture history, and past medical history. Information on current medications including insulin or insulin analog, oral hypoglycemic drugs, and vitamin D and calcium supplements was also collected for each patient.

Moreover, anthropometric data including body weight, height, waist circumference, heart rate, and blood pressure were collected through physical examination.

### Dual-energy X-ray absorptiometry (DXA)

The DXA measurements (Prodigy Advance, GE Lunar Corporation, USA) were performed at the lumbar spine (L1-L4) and right proximal femur (femoral neck, trochanter, and total) according to our standard scanning and analyzing procedure. The in vivo precision of the measurements for the spine, femoral neck, and trochanter was 1.7%, 1.7%, and 2.1%, respectively. The z-scores of aBMD were also calculated. DXA was performed within one week post-interview.

### High-resolution peripheral quantitative computed tomography (HR-pQCT)

HR-pQCT scans (Xtreme CT II; ScancoMedical, Bruttisellen, Switzerland) with an isotropic voxel size of 61 μM at the nondominant ultradistal radius and ultradistal tibia were used to determine standard morphological, vBMD, and micro-architectural parameters according to a previously described protocol [25]. All participants were scanned with the standard human in vivo scanning protocol (60 kVp, 1000 μA, 100 ms integration time). All scans were finished by a specific technologist and analyzed according to the standard manufacturer’s method.

After standard analysis software processing, we obtained the data of the following parameters: total volumetric bone mineral density (Tt.vBMD), cortical vBMD (Ct.vBMD), trabecular vBMD (Tb.vBMD), trabecular bone volume fraction (BV/TV), trabecular number (Tb.N), trabecular thickness (Tb.Th), trabecular separation (Tb.Sp), inhomogeneity of network (=St.Dev of 1/Tb.N (Tb.1/N.SD)), cortical thickness (Ct.Th), intracortical porosity (Ct.Po), and cortical pore diameter (Ct.Po.Dm).

All patients and controls (*n* = 64) underwent HR-pQCT scans and the following standard analysis.

### Statistical analysis

In this study, each T1D patient was classified into one of the two groups according to their age at the time of T1D diagnosis. Those over the age of 18 years at diagnosis were categorized into the adult-onset group, and those younger than 18 at the time of diagnosis were categorized in the juvenile-onset group.

Categorical variables are presented as absolute and relative values (%) and then compared using chi-square tests. Continuous nominal data are expressed as means ± standard deviations (SDs) and were compared using unpaired *t* tests. Continuous non-nominal data are expressed as medians (25th–75th) and compared using Kruskal–Wallis tests (non-nominal).

HR-pQCT data were first compared between each T1D patient and the corresponding control subject, then between each juvenile-onset T1D patient and the corresponding control subject, then between each adult-onset T1D patient and the corresponding control subject, and, finally, between juvenile-onset and adult-onset T1D patients. Because BMI, disease duration, and insulin dose were significantly different between juvenile-onset and adult-onset T1D patients, analysis of covariance (ANCOVA) was performed. Model 1 was adjusted only for BMI, and Model 2 was adjusted for BMI, disease duration, and insulin dose. Because this was a pilot and preliminary study, we aimed to propose a hypothesis for further research. Therefore, we did not formally adjust for multiple comparisons; however, we have interpreted our results with caution. Data were analyzed using SPSS 26.0 (IBM Corp., Armonk, NY, USA); all statistical tests were two-tailed, and *p*-values < 0.05 were considered to indicate statistical significance.

## Results

### Subject characteristics

A total of 32 adult male T1D patients were recruited for this study; 15 of them had juvenile-onset T1D, and the other 17 had adult-onset T1D. The general clinical characteristics of the study population are presented in Table 1. The total study population had a mean age of 34 ± 11years, age at diabetes diagnosis of 22 ± 11years, HbA1c of 7.0%, 25-(OH)D of 20.7 ng/mL, and testosterone level of 5.1 ng/mL. Compared to juvenile-onset patients, adult-onset patients had lower BMIs, lower waist circumferences (20.5 ± 2.2 kg/m^2^ vs. 25.3 ± 2.8 kg/m^2^, *p* = 0.002; and 73.1 ± 5.3 cm vs. 84.2 ± 9.6 cm, *p* = 0.003, respectively), and shorter diabetic durations (median 7 years vs. 11 years, *p* < 0.001), and they also required lower insulin doses to maintain glucose control (33 ± 13 u/day vs. 52 ± 15 u/day, *p* = 0.003). Furthermore, adult-onset patients also had lower ALP levels than juvenile-onset patients (63 ± 15 U/L vs. 76 ± 14 U/L, *p* = 0.037), although there was no significant difference in β-CTX levels between the two groups. There were no significant differences in age, blood pressure, HbA1C, or uACR between the juvenile- and adult-onset patients.

**Table 1:** Comparison of clinical characteristics between patients with adult-onset and juvenile-onset type 1 diabetes

	Total (*n* = 32)	Juvenile onset (*n* = 15)	Adult onset (*n* = 17)	*p* value
Age (y)	34 ± 11 (21–61)	32 ± 8 (21–52)	36 ± 12 (22–61)	0.325
Age at onset (y)	22 ± 11 (2–55)	10 ± 5 (2–17)	29 ± 9 (19–55)	<0.001
Diabetes duration (y)	12 (4.5–19.5)	11 (4–19)	7 (0.5–14)	<0.001
Insulin dose (µ/day)	42 ± 17 (8–66)	52 ± 15 (26–66)	33 ± 13 (8–58)	0.003
Fracture history (*n*, %)	6 (18.8%)	3 (20%)	3 (17.6%)	0.686
Hypertension (*n*, %)	1 (3.1%)	1 (6.7%)	0 (0%)	0.455
Hyperlipidemia (*n*, %)	3 (9.4%)	2 (13.3%)	1 (5.9%)	0.429
Diabetic nephropathy (*n*, %)	3 (9.4%)	2 (13.3%)	1 (5.9%)	0.346
Current smoker (*n*, %)	6 (18.8%)	5 (33.3%)	1 (5.9%)	0.070
Ex-smoker (*n*, %)	1 (3.1%)	1 (6.7%)	0 (0%)	0.474
Alcohol consumption (*n*, %)	3 (9.4%)	2 (13.3%)	1 (5.9%)	0.500
Calcium supplement (*n*, %)	1 (3.1%)	0 (0%)	1 (5.9%)	0.500
Vit D supplement (*n*, %)	0 (0%)	0 (0%)	0 (0%)	0.686
BMI (kg/m^2^)	22.7 ± 3.4 (17.7–30.7)	25.3 ± 2.8 (21.5–30.7)	20.5 ± 2.2 (17.7–24.5)	0.002
WC (cm)	78.7 ± 9.5 (67–105)	84.2 ± 9.6 (68–105)	73.1 ± 5.3 (67–86)	0.003
SBP (mmHg)	118 ± 16 (98–160)	120 ± 12 (100–144)	116 ± 18 (98–160)	0.567
DBP (mmHg)	76 ± 9 (56–96)	79 ± 8.5 (70–96)	74 ± 10 (56–86)	0.246
HR (bpm)	79 ± 10 (64–96)	81 ± 12 (64–96)	79 ± 9 (64–96)	0.603
HbA1C (%)	7.0 ± 1.2 (5.2–10)	7.2 ± 1.5 (5.7–9.2)	7.1 ± 1.2 (5.6–10.0)	0.882
uACR (mg/g Cr)	4.5 (3~11)	9 (3.8–20.1)	3 (3~6)	0.312
Ca (mmol/L)	2.3 ± 0.1 (2.04–2.52)	2.3 ± 0.1 (2.13–2.43)	2.3 ± 0.1 (2.0–2.5)	0.525
P (mmol/L)	1.2 ± 0.2 (0.8–1.7)	1.2 ± 0.2 (0.9–1.5)	1.2 ± 0.2 (0.8–1.7)	0.954
ALP (µ/L)	68 ± 15 (44–102)	76 ± 14 (53–102)	63 ± 15 (44–89)	0.037
β-CTX (ng/mL)	0.44 ± 0.20 (0.11–0.90)	0.44 ± 0.23 (0.11–0.90)	0.45 ± 0.18 (0.2–0.8)	0.889
25-OHD (ng/mL)	20.7 ± 8.3 (7.6–35.8)	21.0 ± 7.5 (10–32.4)	21.2 ± 8.1 (10.3–32.7)	0.963
Testosterone (ng/mL)	5.1 ± 1.7 (2.29–9.47)	5.5 ± 1.6 (3.2–9.5)	4.9 ± 1.5 (2.9–7.8)	0.311

Normally distributed data are expressed as means ± SDs, with minimum and maximum values; non-normally distributed data are expressed as medians (25th–75th)

*BMI* body mass index, *WC* waist circumference, *SBP* systolic pressure, *DBP* diastolic pressure, *uACR* urinary albumin-to-creatinine ratio, *ALP* alkaline phosphatase, *β-CTX* cross linked C-telopeptide of type I collagen, *25OHD* 25-hydroxy vitamin D

A total of 6 patients reported 6 prevalent fractures, all occurring after a fall or trauma, and there was no significant difference in fracture history between the juvenile-onset and adult-onset T1D groups. There were 3 patients with diabetic nephropathy: one case manifested as macroalbuminuria and the other two only as microalbuminuria.

### DXA measurement of spine and hip

There was no significant difference in BMD in the hip between adult-onset and juvenile-onset T1D patients (Table 2). However, adult-onset T1D patients had lower BMDs than juvenile-onset T1D patients at the lumbar spine (L1-L4) (*z* score 0.29 vs. 0.97, p = 0.048; 1.105 ± 0.151 vs. 1.227 ± 0.058 g/cm^2^, *p* = 0.007) (Table 2), but after adjusting for BMI, the difference in BMD at L1-L4 lost significance (data not shown).

**Table 2:** Differences in bone mineral density (BMD) z-scores in type 1 diabetes subjects

BMD	Juvenile onset (*n* = 15)	Adult onset (*n* = 17)	*p* value
Hip			
Neck *z*-score	0.19 (−0.63~1.01)	−0.23 (−1.14~0.70)	0.318
Neck (g/cm^2^)	1.013 ± 0.153	0.932 ± 0.154	0.193
Hip total *z*-score	0.30 (−0.73~1.23)	−0.17 (−1.07~0.79)	0.302
Hip total (g/cm^2^)	1.028 ± 0.174	0.965 ± 0.147	0.322
Spine			
L1–L4 *z*-score	0.97 (0.34~1.20)	0.29 (−0.71~1.29)	0.048
L1–L4 (g/cm^2^)	1.227 ± 0.058	1.105 ± 0.151	0.007

### HR-pQCT measurement of radius and tibia

#### Comparisons between diabetic and control subjects

Tables 3 and 4 show the HR-pQCT related parameters for the radius and the tibia, respectively. At the radius, T1D had lower total and trabecular vBMD (−16.1%, *p* < 0.001; and −21.7%, *p* < 0.001; respectively), lower trabecular BV/TV (−20.7%, *p* < 0.001), lower Tb.Th(−11.5%, *p* < 0.001), and lower Ct.Th (−13.4%, *p* = 0.006) but higher Tb.Sp (+11.3%, *p* = 0.024) than the controls. Subgroup analysis showed similar findings in the adult-onset and juvenile-onset T1D patients compared to related sub-control groups. Adult-onset and juvenile-onset T1D patients both had lower total vBMD (−16.6%, *p* = 0.009; and −14.3%, *p* = 0.006, respectively) and trabecular vBMD (−20.7%, *p* = 0.007; and −23.3%, *p* < 0.001, respectively). They also had lower trabecular BV/TV (−20.7%, *p* = 0.006; and −24.1%, *p* = 0.001, respectively) and Tb.Th (−7.7%, *p* = 0.003; and −15.4%, *p* < 0.001, respectively) than the age- and BMI-matched controls. Specifically, adult-onset T1D patients also demonstrated lower Tb.N (−10.7%, *p* = 0.045) and higher Tb.Sp (+16.1%, *p* = 0.024), while juvenile-onset T1D patients demonstrated lower Ct.Th (−15.0%, *p* = 0.035).

**Table 3:** Unadjusted HR-pQCT derived vBMD and microarchitecture parameters at the ultradistal radius, and differences between adult- versus juvenile-onset type 1 diabetes patients and controls

Ultradistal radius	All controls (*n* = 28)	All T1D (*n* = 28)	Control 1 (*n* = 15)	Juvenile-onset (*n* = 15)	Control 2 (*n* = 17)	Adult-onset (*n* = 17)	*p*1	*p*2	*p*3	*p*4	*p*5	*p*6
Volumetric density												
Tt vBMD (mg/cm^3^)	376.3 ± 60.6	315.9 ± 54.1	389.4 ± 53.7	329.9 ± 35.0	367.8 ± 64.9	306.9 ± 62.8	**<0.001**	**0.006**	**0.009**	0.279	0.476	0.384
Ct vBMD (mg/cm^3^)	913.2 ± 58.1	912.2 ± 43.9	916.5 ± 67.4	925.1 ± 40.4	911.0 ± 53.4	903.9 ± 45.3	0.946	0.720	0.678	0.218	0.463	0.428
Tb vBMD (mg/cm^3^)	195.9 ± 30.9	153.3 ± 39.1	198.1 ± 26.3	151.9 ± 25.8	194.5 ± 34.2	154.3 ± 46.5	**<0.001**	**<0.001**	**0.007**	0.878	0.207	0.066
Microarchitecture												
Tb BV/TV	29.0 ± 4.3	22.7 ± 5.7	29.1 ± 4.0	22.2 ± 3.9	28.9 ± 4.6	23.0 ± 6.8	**<0.001**	**0.001**	**0.006**	0.742	0.179	0.050
Tb. N (1/mm)	1.48 ± 0.21	1.38 ± 0.22	1.46 ± 0.21	1.45 ± 0.20	1.50 ± 0.21	1.34 ± 0.23	0.086	0.886	**0.045**	0.197	0.721	0.713
Tb.Th (mm)	0.26 ± 0.01	0.23 ± 0.02	0.26 ± 0.02	0.22 ± 0.01	0.26 ± 0.01	0.24 ± 0.02	**<0.001**	**<0.001**	**0.003**	**0.031**	0.098	0.064
Tb.Sp (mm)	0.62 ± 0.10	0.69 ± 0.12	0.63 ± 0.09	0.65 ± 0.10	0.62 ± 0.10	0.72 ± 0.12	**0.024**	0.524	**0.024**	0.164	0.871	0.902
1/Tb.N.SD (mm)	0.25 ± 0.05	0.27 ± 0.05	0.25 ± 0.04	0.24 ± 0.04	0.25 ± 0.06	0.28 ± 0.05	0.183	0.881	0.104	**0.032**	0.890	0.899
Ct.Th (mm)	1.27 ± 0.25	1.10 ± 0.21	1.33 ± 0.23	1.13 ± 0.18	1.24 ± 0.25	1.08 ± 0.22	**0.006**	**0.035**	0.062	0.520	0.357	0.256
Ct.Po (%)	0.63 ± 0.43	0.60 ± 0.43	0.68 ± 0.59	0.53 ± 0.21	0.59 ± 0.29	0.65 ± 0.52	0.828	0.426	0.690	0.460	**0.039**	0.070
Ct.Po.Dm (mm)	0.18 ± 0.02	0.17 ± 0.03	0.18 ± 0.02	0.16 ± 0.02	0.18 ± 0.02	0.17 ± 0.03	0.150	0.082	0.595	0.221	0.148	0.164

Data are expressed as means ± SDs. Significant values are shown in bold. *p*1: All T1D patients vs. all controls. *p*2: Juvenile-onset patients vs. control 1. *p*3: Adult-onset patients vs. control 2. *p*4: Juvenile-onset patients vs. adult-onset patients, unadjusted. *p*5: Juvenile-onset patients vs. adult-onset patients, adjusted for BMI only. *p*4: Juvenile-onset patients vs. adult-onset patients, adjusted for BMI, disease duration, and insulin dose. T1D: type 1 diabetes; Control 1: control subjects matched for age, sex, and BMI for juvenile-onset T1D patients; Control 2: control subjects matched for age, sex, and BMI for adult-onset T1D patients

*vBMD* volumetric bone mineral density, *Tt.vBMD* total volumetric bone mineral density, *BV/TV* trabecular bone volume fraction, *Tb.vBMD* trabecular vBMD, *Tb.N* trabecular number, *Tb.Th* trabecular thickness, *Tb.Sp* trabecular separation, *Ct.vBMD* Cortical vBMD, *1/Tb.N.SD* st. dev of 1/Tb.N, *Ct.Th* cortical thickness, *Ct.Po* intracortical porosity, *Ct.Po.Dm* cortical pore diameter, *HR-pQCT* high-resolution peripheral quantitative computed tomography

**Table 4:** Unadjusted HR-pQCT derived vBMD and microarchitecture parameters at the ultradistal tibia, and differences between adult- versus juvenile-onset T1D patients and controls

Ultradistal tibia	All Controls (*n* = 28)	All T1D (*n* = 28)	Control 1 (*n* = 15)	juvenile-onset (*n* = 15)	Control 2 (*n* = 17)	adult-onset (*n* = 17)	*p*1	*p*2	*p*3	*p*4	*p*5	*p*6
Volumetric density												
Tt vBMD (mg/cm^3^)	335.5 ± 42.5	293.8 ± 63.2	338.5 ± 41.3	308.5 ± 38.2	333.5 ± 44.4	285.1 ± 73.8	**0.006**	0.101	**0.027**	0.364	0.548	0.448
Ct vBMD (mg/cm^3^)	936.5 ± 35.4	913.1 ± 54.1	929.7 ± 42.4	934.0 ± 32.5	940.9 ± 30.8	900.8 ± 61.0	0.062	0.801	**0.021**	0.077	0.256	0.355
Tb vBMD (mg/cm^3^)	187.6 ± 32.8	168.9 ± 51.0	194.0 ± 33.3	172.2 ± 34.7	183.5 ± 32.8	167.0 ± 59.5	0.111	0.158	0.325	0.805	0.346	0.255
Microarchitecture												
Tb BV/TV	28.0 ± 4.2	25.5 ± 6.6	28.9 ± 4.4	25.9 ± 4.8	27.5 ± 4.2	25.3 ± 7.6	0.099	0.15	0.316	0.852	0.329	0.217
Tb. N (1/mm)	1.30 ± 0.20	1.35 ± 0.26	1.34 ± 0.26	1.53 ± 0.24	1.28 ± 0.16	1.24 ± 0.21	0.447	0.09	0.564	**0.003**	0.750	0.648
Tb.Th (mm)	0.28 ± 0.02	0.26 ± 0.03	0.27 ± 0.02	0.24 ± 0.01	0.28 ± 0.02	0.26 ± 0.03	**0.003**	**<0.001**	0.262	**0.004**	0.136	0.220
Tb.Sp (mm)	0.75 ± 0.11	0.74 ± 0.16	0.74 ± 0.14	0.63 ± 0.11	0.76 ± 0.10	0.80 ± 0.17	0.705	0.068	0.356	**0.002**	0.807	0.812
Tb.1/N.SD (mm)	0.32 ± 0.06	0.30 ± 0.07	0.31 ± 0.07	0.25 ± 0.05	0.33 ± 0.06	0.33 ± 0.06	0.299	0.023	0.733	**0.001**	0.911	0.741
Ct.Th(mm)	1.67 ± 0.28	1.46 ± 0.34	1.69 ± 0.27	1.52 ± 0.22	1.65 ± 0.29	1.43 ± 0.39	**0.018**	0.138	**0.068**	0.505	0.376	0.436
Ct.Po (%)	1.97 ± 0.81	2.39 ± 1.27	2.15 ± 0.95	1.85 ± 0.64	1.86 ± 0.71	2.71 ± 1.44	0.147	0.42	**0.036**	**0.044**	**0.025**	0.121
Ct.Po.Dm (mm)	0.23 ± 0.03	0.24 ± 0.04	0.25 ± 0.04	0.23 ± 0.04	0.23 ± 0.03	0.24 ± 0.04	0.859	0.501	0.392	0.792	0.097	0.061

Data are expressed as means ± SDs. Significant values are shown in bold. *p*1: All T1D vs. all controls. *p*2: Juvenile-onset patients vs. control 1. *p*3: Adult-onset patients vs. control 2. *p*4: Juvenile-onset patients vs. adult-onset patients, unadjusted. *p*5: Juvenile-onset patients vs. adult-onset patients, adjusted for BMI only. *p*4: Juvenile-onset patients vs. adult-onset patients, adjusted for BMI, disease duration, and insulin dose. T1D: type 1 diabetes; Control 1: control subjects matched for age, sex, and BMI for juvenile-onset T1D patients; Control 2: control subjects matched for age, sex, and BMI for adult-onset T1D patients

*vBMD* volumetric bone mineral density, *Tt.vBMD* total volumetric bone mineral density, *BV/TV* trabecular bone volume fraction, *Tb.vBMD* trabecular vBMD, *Tb.N* trabecular number, *Tb.Th* trabecular thickness, *Tb.Sp* trabecular separation, *Ct.vBMD* cortical vBMD, *1/Tb.N.SD* st.dev of 1/Tb.N, *Ct.Th* cortical thickness, *Ct.Po* intracortical porosity, *Ct.Po.Dm* cortical pore diameter, *HR-pQCT* high-resolution peripheral quantitative computed tomography

At the tibia, T1D patients had lower total vBMD (−12.4%, *p* = 0.006), lower Tb.Th (−7.1%, *p* = 0.003), and lower Ct.Th (−12.6%, *p* = 0.018) than the controls. Adult-onset T1D patients had lower total vBMD (−14.5%, *p* = 0.027), lower Ct vBMD (−4.3%, *p* = 0.021), and higher Ct.Po (+42.1%, *p* = 0.036) than age- and BMI-matched controls. Juvenile-onset T1D patients had lower Tb.Th (−11.1%, *p* < 0.001) and Tb.1/N.SD (−19.4%, *p* = 0.023) than the age- and BMI-matched controls.

#### Comparisons between adult-onset and juvenile-onset T1D patients

Juvenile-onset T1D patients had lower Tb.Th (−8.3%, *p* = 0.031) and 1/Tb.N.SD (−14.3%, *p* = 0.023) at the radius than adult-onset T1D patients. After correction for BMI only, those differences lost significance, but Ct.Po was found to be significantly higher in the adult-onset group than in the juvenile-onset group (+22.6%, *p* = 0.039). After further correction for BMI, disease duration, and insulin dose, there was a trend toward lower trabecular vBMD (−1.6%, *p* = 0.066), BV/TV (−4.3%, *p* = 0.05), and Tb.Th (−8.3%, *p* = 0.064) in juvenile-onset T1D patients, and the difference for higher Ct.Po in the adult-onset group became borderline significant (+22.6%, *p* = 0.070).

At the tibia, juvenile-onset T1D patients had higher Tb.N (+23.4%, *p* = 0.003), lower Tb.Th (−7.7%, *p* = 0.004), lower Tb.Sp (−21.3%, *p* = 0.002), and lower 1/Tb.N.SD (−24.2%, *p* = 0.001) than adult-onset patients, while adult-onset patients had higher Ct.Po (+46.5%, *p* = 0.044) and a trend toward lower Ct vBMD (−3.6%, *p* = 0.077) than juvenile-onset patients. After correction for BMI only, those differences lost significance, with the exception of Ct.Po. This value remained significantly different (+22.6%, *p* = 0.025). After further correction for BMI, disease duration, and insulin dose, none of these differences persisted, but only a trend toward higher Ct.Po.Dm (+4.3%, *p* = 0.061) was found in adult-onset patients.

### Partial correlation results

After controlling for BMI, no significant relationship was found between HbA1C, uACR, 25OHD, insulin dose, or testosterone level with DXA- and HR-pQCT-related parameters.

## Discussion

Herein, we report the first study describing HR-pQCT derived bone microarchitecture in Asian men with T1D. We found that males with both adult-onset and juvenile-onset T1D exhibited deficits in volumetric density and microarchitecture when compared with control subjects. Interestingly, we also found differences between the two age-categorized groups; the adult-onset group tended to suffer more prominent cortical deficits (particularly higher porosity), while the juvenile-onset group tended to display a greater degree of trabecular deficits.

Previous evidence regarding cortical microarchitecture in Asian T1D patients is lacking. Our results were consistent with those from the only previous study to demonstrate lower cortical CSA and thickness in young and middle-aged Japanese T1D adults [18]. Two studies conducted in a Western population with older age and longer disease duration also found similar results. One found that T1D patients presented with smaller cortical thickness at the distal radius [26], while the other found that the lower radius Ct.Th was more profound in T1D patients with MVD [20]. Research findings are contradictory with regard to children and adolescents with T1D. Some studies found these patients to have smaller CSA and thinner Ct.Th when compared with controls [27–31], while others did not [32–34]. These differences may be due to the age of patients studied. One study only found smaller total and cortical CSA in patients of prepuberty and early puberty ages but not in adolescents [35]. Another longitudinal study with a longer follow-up period found that T1D children with manifestation at an early age had transient impaired bone development, while these patients had a normal bone size in adolescence [34].

Besides lower cortical thickness, we have also found T1D male patients to have high cortical porosity in the tibia, especially in the adult-onset group. One previous study in adults with T1D did not observe an increase in cortical porosity [20], but other studies conducted among T2D patients found similar results [16, 36, 37]. In a recent study, female T1D patients aged 10–16 years also had higher cortical porosity measured by HR-pQCT, particularly those with HbA1c >8.5%, which was higher than that in our patients [17].

Furthermore, we found that T1D patients also exhibited deficits in trabecular microarchitecture, with lower BV/TV, Tb.Th, Tb.N, and homogeneity and higher Tb.Sp than control subjects. There is no previous study regarding trabecular microarchitecture in Asian T1D patients, but our results were consistent with studies on Western populations. One previous study found lower BV/TV in T1D adults with MVD [20]; however, few patients in our study had evident MVD. Another study using MRI also found lower BV/TV and higher Tb.Sp in adult T1D patients [38]. Studies on adolescents also found lower BV/TV, lower Tb.N and higher Tb.Sp than control subjects [17, 39]. Only one study has reported no abnormalities in trabecular bone histomorphometric or μCT variables in a small cohort of adult T1D patients [40].

In addition, we found that both adult-onset and juvenile-onset T1D patients demonstrated lower vBMD, thinning of the trabeculae, and cortical thickness in adulthood when compared with controls. This is consistent with studies reporting that women with T1D exhibited significant BMD differences in the post-teenage years [12] and that adult T1D patients demonstrated reduced BMD at the time of disease onset [41]. Together, these findings may provide epidemiological evidence that the incident fracture risk in T1D begins in childhood and increases across the lifespan [1] and therefore should be taken into consideration as early as possible.

Last, our study also conducted comparisons between adult-onset and juvenile-onset male T1D patients. We found that the adult-onset group tended to suffer more prominent cortical deficits, particularly porosity, while the juvenile-onset group tended to have a greater degree of trabecular deficits. Only one recent study conducted similar comparisons; it revealed that T1D onset in women before the age of 20 was associated with lower vBMD and reduced cortical thickness [24]. The mechanisms underlying bone-parameter diversity between juvenile-onset and adult-onset T1D were difficult to explain. TID is a well-known cause of secondary osteoporosis, and the pathogenesis of fracture seems to be multifactorial, depending on hyperglycemia, insulin/IGF-1 deficiency, hypogonadism, kidney disease, and vitamin D deficiency [42]. However, there was no significant difference in HbA1C, uACR, 25OHD, or serum testosterone concentration between these two groups in the present study. Thus, age at T1D onset could be an important influential factor for incident fracture risk, and future studies are necessary to further investigate the mechanisms involved.

Our study had some limitations that require consideration. First, this was a pilot and cross-sectional study that was unable to expose a cause-effect relationship. However, it may propose hypotheses for further research. Second, the relatively small sample size limited the precision of our results. Larger studies will be required to confirm these results. Third, the control group did not undergo DXA to generate aBMD data. Finally, we did not perform HR-pQCT derived micro-finite element analysis to evaluate bone mechanical property.

## Conclusions

We found that Asian males with both adult-onset and juvenile-onset T1D exhibit deficits in vBMD and microarchitecture when compared with controls, and differences exist in cortical and trabecular microarchitecture between the two groups. To the best of our knowledge, this is the first study conducted in Asian males with T1D using HR-pQCT to evaluate detailed bone microarchitecture. Further studies with larger sample populations are necessary to further confirm our findings and explore their clinical applications with respect to T1D patients.

## Data Availability

All data generated or analyzed during this study were included in the article.
